# ^223^Ra-dichloride therapy of bone metastasis: optimization of SPECT images for quantification

**DOI:** 10.1186/s13550-019-0488-7

**Published:** 2019-02-21

**Authors:** Nadia Benabdallah, Michela Bernardini, Marta Bianciardi, Claire de Labriolle-Vaylet, Didier Franck, Aurélie Desbrée

**Affiliations:** 1 Internal Dose Assessment Laboratory, Institute for Radiological Protection and Nuclear Safety (IRSN), Fontenay-aux-Roses, France; 2 Nuclear Medicine Department, European Hospital George Pompidou (HEGP), Paris, France; 3 Department of Radiology, Athinoula A. Martinos Center for Biomedical Imaging, Massachusetts General Hospital and Harvard Medical School, Boston, USA; 4 UPMC, Univ Paris 06 Biophysics, Paris, France; 5 Nuclear Medicine Department, Trousseau Hospital, Paris, France

**Keywords:** Bone metastasis, ^223^Ra, Radionuclide therapy, Quantitative imaging, SPECT imaging, Physical phantoms

## Abstract

**Background:**

^223^Ra imaging is crucial to evaluate the successfulness of the therapy of bone metastasis of castration-resistant prostate cancer (CRPC). The goals of this study were to establish a quantitative tomographic ^223^Ra imaging protocol with clinically achievable conditions, as well as to investigate its usefulness and limitations.

We performed several experiments using the Infinia Hawkeye 4 gamma camera (GE) and physical phantoms in order to assess the optimal image acquisition and reconstruction parameters, such as the windows setting, as well as the iteration number and filter of the reconstruction algorithm. Then, based on the MIRD pamphlet 23, we used a NEMA phantom and an anthropomorphic TORSO® phantom to calibrate the gamma camera and investigate the accuracy of quantification.

**Results:**

Experiences showed that the 85 keV ± 20%, 154 keV ± 10%, and 270 keV ± 10% energy windows are the most suitable for ^223^Ra imaging.

The study with the NEMA phantom showed that the OSEM algorithm with 2 iterations, 10 subsets, and the Butterworth filter offered the best compromise between contrast and noise. Moreover, the calibration factors for different sphere sizes (26.5 ml, 11.5 ml, and 5.6 ml) were constant for ^223^Ra concentrations ranging between 6.5 and 22.8 kBq/ml. The values found are 73.7 cts/s/MBq, 43.8 cts/s/MBq, and 43.4 cts/s/MBq for 26.5 ml, 11.5 ml, and 5.6 ml sphere, respectively. For concentration lower than 6.5 kBq/ml, the calibration factors exhibited greater variability pointing out the limitations of SPECT/CT imaging for quantification.

By the use of a TORSO® phantom, we simulated several tumors to normal tissue ratios as close as possible to clinical conditions. Using the calibration factors obtained with the NEMA phantom, for ^223^Ra concentrations higher than 8 kBq/ml, we were able to quantify the activity with an error inferior to 18.8% in a 5.6 ml lesion.

**Conclusions:**

Absolute quantitative ^223^Ra SPECT imaging appears feasible once the dimension of the target is determined. Further evaluation should be needed to apply the calibration factor-based quantitation to clinical ^223^Ra SPECT/CT imaging. This will open the possibility for patient-specific ^223^Ra treatment planning and therapeutic outcome prediction in patients.

## Background

^223^Ra-dichloride (Xofigo®, Bayer HealthCare) is the first alpha particle emitter that has received marketing authorization from European Commission and approval by the US Food and Drug Administration for the treatment of patients with castration-resistant prostate cancer (CRPC) metastasized to bones. Since its clinical approval in 2013, more than 27,000 patients have received Xofigo® worldwide [1]. This radiopharmaceutical is currently involved in 45 clinical trials [2]. In the UK, it was used in 95% of all radiopharmaceutical-based treatments of bone metastasis in 2015 [3]. The total number of patients with bone metastasis treated with radiopharmaceuticals has increased by nearly 400% from 2007 to 2015 due to ^223^Ra use.

Several studies have now shown solid evidence of the clinical utility of ^223^Ra for patients with CRPC [4–11]. In order to improve the patient-specific treatment, images of the distribution of ^223^Ra in the patient body and image-based dosimetry are needed. Indeed, these images will allow to ascertain whether ^223^Ra uptakes correspond to bone lesion locations and to predict any toxicity in the organs at risk. Furthermore, with an adequate calibration, these images can be used to better assess the tolerance dose of bone marrow and to correlate the absorbed dose to bone lesion responses.

There is a growing literature on this complex topic. Despite a low detectability due to a low-injected activity, ^223^Ra emits useful photons with a probability of emission that enables their detection by a gamma camera [12]. For instance, gamma-camera-based planar imaging of ^223^Ra was shown to be feasible in in vivo studies allowing biodistribution and pharmacokinetics investigation in metastatic prostate cancer [13]. However, recent work [14] showed that not all the metastatic lesions detected on bone scintigraphy images are visible on planar ^223^Ra images. Interestingly, the feasibility of quantitative ^223^Ra planar imaging with a gamma camera was also reported [15–18]. Studies on ^223^Ra planar imaging showed good results and allowed to obtain an activity quantification within 20% error [14, 16, 19, 20]. However, the main limitation of ^223^Ra quantification is the difficulty to precisely segment lesions on planar images, because lesions often superimpose with regions of no interest [16]. For instance, in the case of prostate cancer, the detection and quantitative investigation of bone metastasis (often occurring in the pelvis and the lumbar spine) in planar images is complex because of its overlap with other structures, such as the intestine [13]. In addition, lesions are sometimes visible only in the anterior or posterior view, which limits the quantification of ^223^Ra activity [14]. Further, the improved quantitative accuracy of 3D tomographic imaging modalities over 2D planar imaging is well established [21–23]; yet only one study so far investigated the feasibility and usefulness of single-photon emission computed tomography (SPECT) semi-quantitative imaging for ^223^Ra [24]. In [24], Owaki et al. demonstrated the advantage of SPECT imaging on separating pathological bone uptakes from bowel uptakes. This result suggests that quantitative SPECT/CT ^223^Ra imaging is a promising method able to overcome the limitations of planar imaging and to better assess individual lesions.

In this study, we aimed at thoroughly characterizing ^223^Ra quantitative SPECT imaging of bone metastasis. In SPECT imaging, careful attention is needed to compensate for image degrading factors and to calibrate the system. Consequently, we optimized the acquisition and reconstruction parameters for SPECT ^223^Ra imaging. A calibration method for quantitative SPECT ^223^Ra imaging was also implemented, and the limitations analyzed. First, measurements were performed to evaluate the imaging sensitivity and spatial resolution, as well as to optimize the energy window setting. Then, a physical phantom was used to assess the optimal reconstruction parameters for quantification purposes. Finally, we investigated the accuracy of quantitative imaging and its limitations by applying these parameters to SPECT ^223^Ra imaging of an anthropomorphic phantom.

## Methods

### Energy windows setting

An Infinia Hawkeye 4 gamma camera (General Electric, USA), equipped with a 5/8-in. crystal and a medium-energy general-purpose (MEGP) collimator, was used. Note that Owaki et al. [24] proved in a clinical study that a high-energy general-purpose (HEGP) collimator gives a higher image contrast than a MEGP collimator, yet an HEGP collimator was not available in our study. The energy window setting was chosen based on the maximum intensities of photons emitted by ^223^Ra. Therefore, three photopeak energy windows were chosen: 85.0 keV ± 20%, 154.0 keV ± 10%, and 270.0 keV ± 10. This choice includes several photon emission ranges of ^223^Ra and the 271.0 keV photons emitted from daughter products ^219^Rn. Then, three energy windows for scatter correction were chosen: 47.0 keV to 67.0 keV, 103.0 keV to 123.0 keV, and 210.2 keV to 242.8 keV.

In order to quantify the contribution of each of these energy windows, we used first the NEMA phantom (Data Spectrum™, USA). This phantom contained six fillable spheres of different diameters: 10, 13, 17, 22, 28, and 37 mm (0.5, 1.1, 2.6, 5.6, 11.5, 26.5 ml). We filled each of these spheres with a solution of 2.7 kBq/ml of ^223^Ra. In order to model the attenuation and scatter, the remaining portion of the phantom was filled with water. We placed the phantom on the table in the center of the field of view and positioned the gamma camera heads at 10 cm in front of it. We acquired SPECT/CT images. The acquisition parameters were 6° between each projection, 128 × 128 matrix, pixel size 4.4 mm, and circular orbit. For each energy window, the images were reconstructed on the clinical workstation XELERIS (General Electric, USA) using the OSEM algorithm with the following parameters: 2 iterations, 10 subsets, Butterworth filter with *f*_cut_ = 0.48 cycle/cm and *p* = 10. During the reconstructions, each image was corrected for attenuation using CT-based attenuation maps and for scatter using the Jaszczak method [25]. Indeed, during the SPECT/CT acquisition, three images corresponding to the three emission energy windows and three images corresponding to the scatter windows were created. The software also generates three CT-based attenuation maps with attenuation coefficients corresponding to each photopeak energy. So, the projections corresponding to the 85 keV ± 20% emission window were corrected for attenuation with its corresponding attenuation map (μ = 0.179 cm^−1^ in water) and for scatter with the 47–67 keV scatter acquisition. The projections corresponding to the 154 keV ± 10% emission window were corrected for attenuation with its corresponding attenuation map (μ = 0.147 cm^−1^ in water) and for scatter with the 103–123 keV scatter acquisition. Finally, the projections corresponding to the 270 keV ± 10% emission window were corrected for attenuation with its attenuation map (μ = 0.121 cm^−1^ in water) and for scatter with the 210.2–242.9 keV scatter acquisition. Finally, the reconstructed images were summed to evaluate every combination: 85 keV versus 85 keV + 154 keV versus 85 keV + 154 keV + 270 keV.

**Fig. 1:**
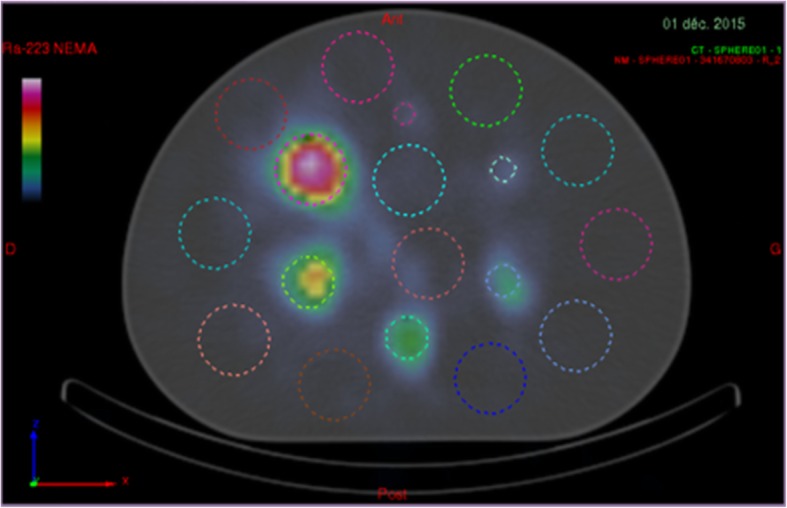
Example of an axial slice of a reconstructed image of the NEMA phantom merged with the CT, showing the ROIs corresponding to the hot spheres, as well as the 37-mm-diameter background ROIs

For the analysis of the background and of the reconstructed signal, we manually delineated six spherical regions of interest (ROIs) using the PLANETOnco software (Dosisoft, France) on the merged SPECT/CT images covering the six hot spheres. The ROI diameters were equal to the physical inner diameters of the six hot spheres. To compare a background noise metric common to all the six hot spheres, we selected as target slice for background analysis an imaging slice containing all the six hot spheres. For each size of the hot spheres, we positioned 12 background 3D ROIs on the target slice. Six different ROI sizes were used, and the ROI diameters were equal to the physical inner diameters of the hot spheres. Figure 1 shows the 37-mm-diameter ROIs (corresponding to the biggest sphere of the NEMA phantom) overlaid on the target slice.

From these measurements, we computed the sensitivity and the signal-to-noise ratio (SNR) for each hot sphere. The sensitivity was calculated as *S* = Counts/(*t A*_0_), where Counts is the total number of counts measured in a ROI within the radioactive volume; *t* is the acquisition duration (seconds); and *A*_*0*_ is the activity in the sphere (MBq). The SNR is defined for each hot sphere as

$$ \mathrm{SNR}=\frac{C_{\mathrm{hot}}}{C_{\mathrm{background}}} $$

where *C*_hot_ is the counts measured in the hot sphere and *C*_background_ is the mean of the counts measured in every background ROIs of the same size as the considered hot sphere.

Second, in order to evaluate the contribution of each emission window on the spatial resolution, we used a Triple Line phantom (Data Spectrum™, USA). This phantom contained three parallel linear capillaries (1 mm diameter): a central one and two lateral ones. These three linear capillaries were filled with 10.8 MBq/ml of ^223^Ra. The phantom was positioned in the center of the field of view at a distance of 10 cm from the heads of the gamma camera. Acquisition and reconstruction parameters were identical to those used for the NEMA phantom. The acquisitions were performed with and without an attenuation medium (water) in the Triple Line phantom. For each acquisition, we fitted the profile of each linear source on both the sagittal and axial views with a Gaussian function. The spatial resolution was calculated as the full width at half maximum (FWHM) of the fitted Gaussian function.

### Reconstruction parameters for SPECT imaging

First, the NEMA phantom was used to assess the best reconstruction for quantification purposes. Each sphere of the phantom was filled with a solution of 20 kBq/ml of ^223^Ra. The remaining portion of the phantom was filled with water. We placed the phantom on the table in the center of the field of view at a distance of 10 cm from the gamma camera heads. The acquisition parameters were 30 s/projections, 6° between each projection (128 × 128 matrix, pixel size 4.4 mm, and circular orbit).

In order to assess the best filter and number of iterations of the OSEM algorithm, we performed several reconstructions of the SPECT/CT acquisition on the clinic workstation. We tested several configurations, namely, the use of 1 to 10 iterations, with Hann (*f*_cut_ = 1.56 cycle/cm) filter, Butterworth (*f*_cut_ = 0.48 cycle/cm and *p* = 10) filter, Gauss (FWHM = 4 mm) filter, and with no filter (none). The scatter was compensated in each emission windows using the Jaszczak method [25]. Compensation for attenuation was performed using the attenuation maps from the CT acquisition.

In order to study the reconstructed signal, we used the manually delineated ROIs as described in the previous section. The sensitivity and the signal-to-noise ratio (SNR) were calculated for each hot sphere.

Second, the spatial resolution was evaluated with a variable number of iteration (*n* = 1–9). The same Triple Line phantom with the background filled with water as described in Section 2.2 was used. Image acquisition for SPECT was the same as those for the NEMA phantom.

### Calibration factors

We performed SPECT/CT acquisitions on the NEMA phantom over a ^223^Ra concentration range from 1.8 to 22.8 kBq/ml in each sphere. The acquisitions and reconstructions were carried out following the acquisition and reconstruction parameters determined above.

For each ^223^Ra concentration, the calibration factor (CF) (cts/s/MBq) was calculated for each sphere with the following equation [26]:

$$ \mathrm{CF}=\frac{C_{\mathrm{measured}}}{A\times t} $$

where *C*_measured_ is the measured number of counts in the delineated 3D ROI surrounding each hot sphere; *A* denotes the activity in each sphere; and *t* is the acquisition duration.

### Validation with an anthropomorphic phantom

**Fig. 2:**
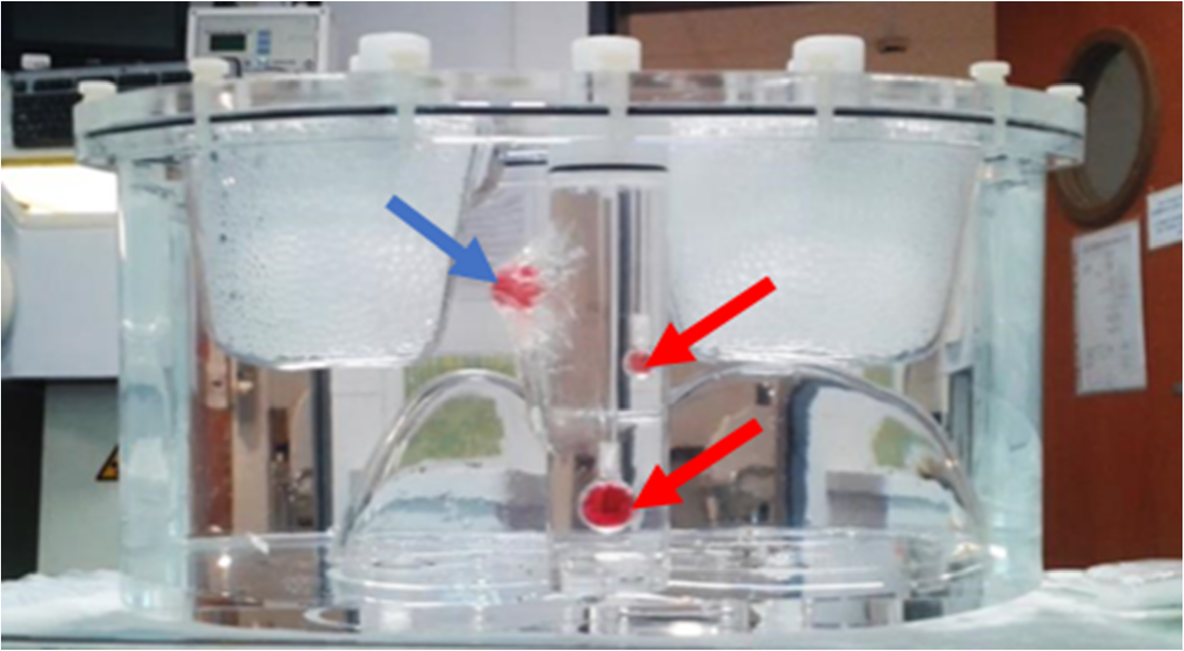
Picture of the TORSO phantom (lateral view) containing two spheres of 0.5 ml and 5.6 ml (indicated with red arrows) inside the cylindrical insert and another sphere of 5.6 ml fixed on it (indicated with a blue arrow)

Once the optimal acquisition and reconstruction parameters were established, the accuracy of quantitative ^223^Ra SPECT imaging was investigated using an anthropomorphic TORSO® phantom (Orion, France), which is designed to mimic as close as possible clinical conditions [27]. This phantom contained a liver insert, lung inserts, and a cylindrical insert of 156 ml nominal volume. Two spheres of 0.5 ml and 5.6 ml were placed in the cylindrical insert and another 5.6 ml sphere was fixed on it (Fig. 2).

In order to mimic ^223^Ra uptakes in the healthy bone and lesions, we used the following tumor to normal tissue (TNT) ratios between the spheres and the cylindrical insert: 6, 10, and 30. The liver insert and the phantom background were filled with water. We filled the lung inserts with Styrofoam® beads and water to mimic lung tissues. Several concentration ranges were used in the spheres: from 2.3 kBq/ml to 8.1 kBq/ml for a TNT = 30, from 8.7 kBq/ml to 21.5 kBq/ml for a TNT = 10, and from 22.8 kBq/ml to 64.0 kBq/ml for a TNT = 6.

To study the accuracy of activity quantification in SPECT/CT images, we used the recovery factor (RF) as described in the MIRD pamphlet 23 [26]. This factor is defined as the ratio between ^223^Ra activity estimated from the image and the true activity in the object. The activity estimated from the image (*A*_expected_) was assessed for each sphere using the following equation:

$$ {A}_{\mathrm{expected}}=\frac{C_{\mathrm{measured}}}{\mathrm{CF}\times t} $$

where *C*_measured_ is the number of counts measured in each spherical ROI corresponding to a hot sphere, CF is the calibration factor, which depends on the sphere sizes and was established on the NEMA phantom, and *t* is the acquisition duration. The recovery factor was analyzed as a function of ^223^Ra concentrations and TNT ratios.

## Results

### Energy windows setting

**Fig. 3:**
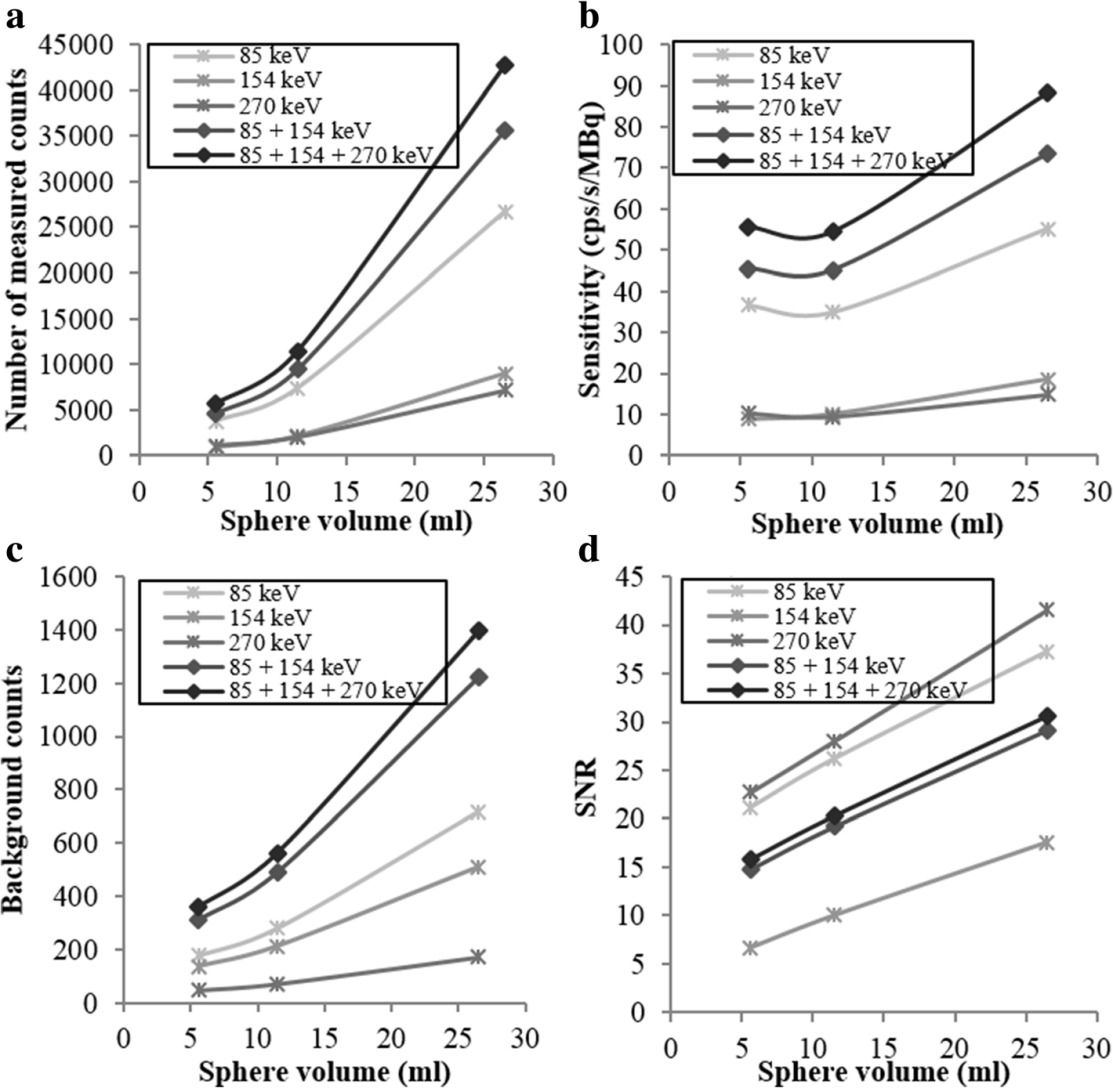
(**a**) Number of measured counts in the hot spheres, (**b**) sensitivity, (**c**) number of counts measured in the background and (**d**) signal-to-noise ratio versus the sphere volume of the NEMA phantom. In each panel, we employed a 30 s/projection acquisition duration, and we also inspected the impact of using images obtained using single energy windows and images obtained by adding the images of different energy windows

The results on the three biggest spheres of the NEMA phantom are shown in Fig. 3. As expected, the number of counts detected in each sphere on the sum of the images obtained with the three selected emission windows exceeded that obtained using only one (85 keV ± 20%) emission window or the sum of two (85 + 154 keV) emission windows by about 35.9% and 17.4%, respectively (Fig. 3a, b). However, the use of three emission windows also increased the background counts as shown in Fig. 3c. This results in lower SNR values on the sum of the images obtained with the three selected emission windows than on the image obtained using only one (85 keV ± 20%) emission window (Fig. 3d). Nevertheless, considering that the biggest challenge of ^223^Ra imaging is the limited number of emitted photons, in order to improve the image sensitivity, we chose to use the sum of the reconstructed images acquired on each energy windows for the evaluation of SPECT/CT reconstruction and quantification. The SNR associated with the 270 keV energy window is higher than the SNR associated with the 85 keV window because of the choice of scatter windows. The scatter window corresponding to the 270 keV energy (210.2 keV to 242.8 keV) includes scatter photons with high intensity. This leads to a small amount of background counts in the reconstructed SPECT image which are mainly scattered and penetrated photons.

Then, the influence of the different energy windows on the spatial resolution was evaluated using the Triple Line phantom. For each attenuation medium and on both axial and sagittal view, the variation between the spatial resolutions of each source was not significant (results not showed). Thus, the spatial resolution was considered constant in the field of view, and each result in Table 1 represents the mean of the FHWM measured on the three linear sources on a selected view.

The contributions of the different energy windows on the spatial resolution are shown in Table 1. The FWHM was measured on the reconstructed images corresponding to each energy windows and on their sum. Except for the spatial resolution on the axial view without attenuation, the spatial resolution was slightly better with the sum of the energy windows than with only the 85 keV ± 20% energy window. This result supports the use of three emission windows over the use of only one (85 keV ± 20%) window.

**Table 1:** Spatial resolutions measured on the reconstructed images obtained with the 85 keV ± 20% energy window and on the sum of the images obtained with each energy window (85 keV ± 20%, 154 keV ± 10%, 270 keV ± 10%)

View	FWHM (mm) without attenuation			FWHM (mm) with attenuation		
	85 keV	Sum	Difference (%)	85 keV	Sum	Difference (%)
Axial	15.8	16.3	3.6	17.2	17.1	− 1.0
Sagittal	16.3	16.0	− 1.5	17.6	16.5	− 6.9

### Reconstruction parameters of SPECT imaging

**Fig. 4:**
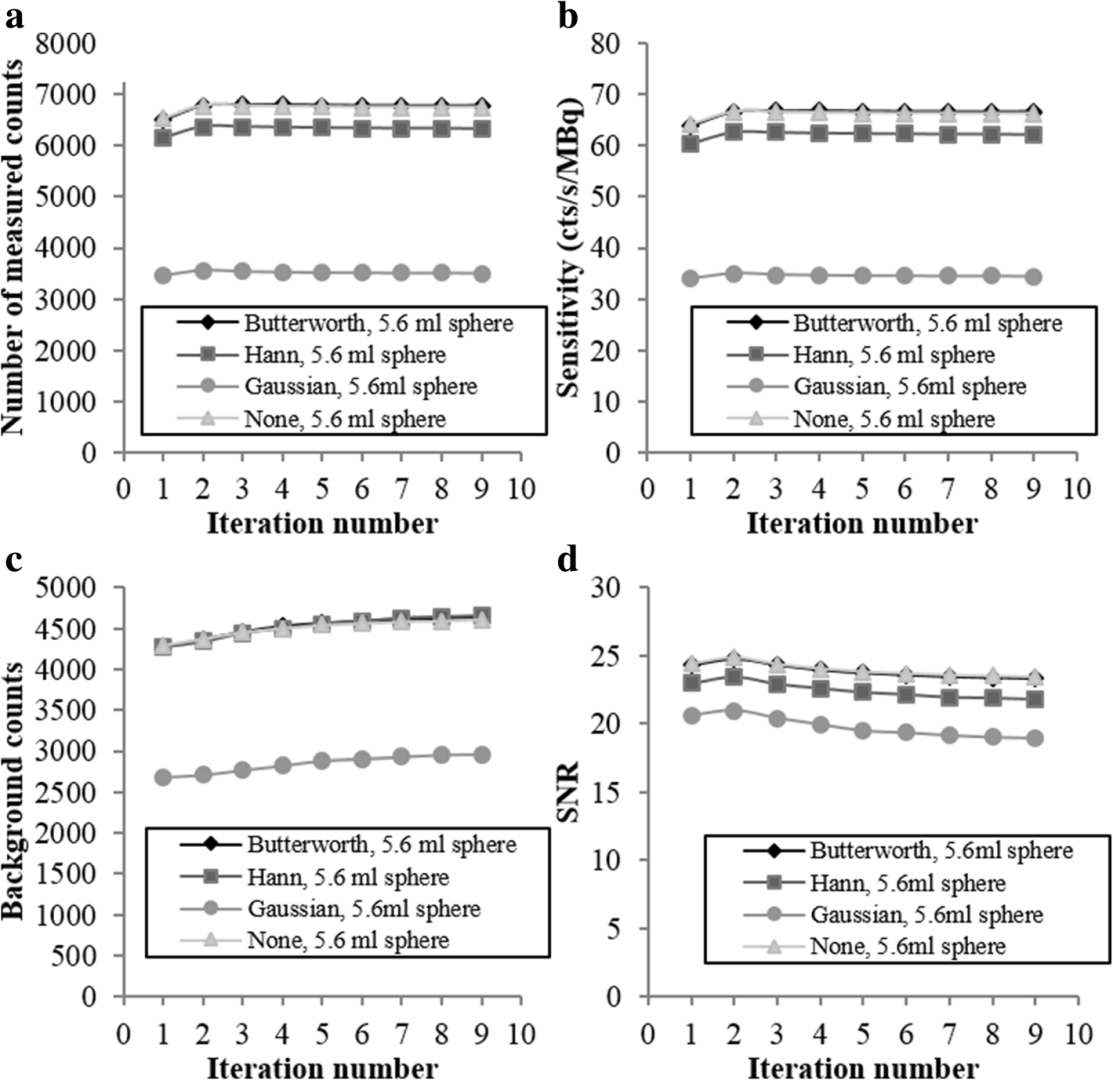
Number of measured counts (**a**), sensitivity (**b**), number of counts measured in the background (**c**), and signal-to-noise ratio (**d**) plotted against iteration number with the ROI fixed at 5.6 ml

**Fig. 5:**
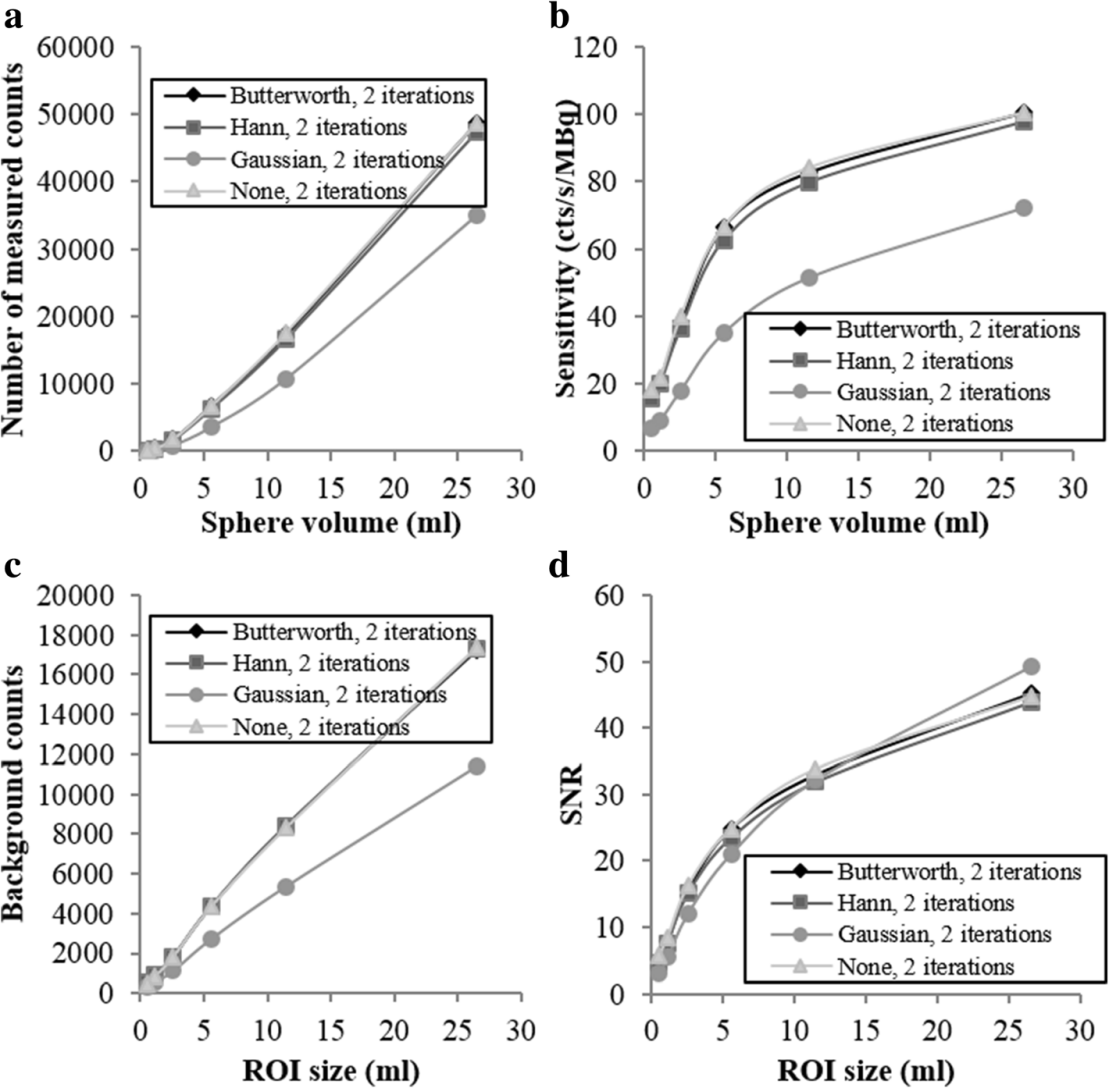
Number of measured counts (**a**), sensitivity (**b**), number of counts measured in the background (**c**), and signal-to-noise ratio (**d**) plotted versus ROI size at fixed iteration 2

Figures 4 and 5 present the results for the sensitivities, the SNR, and the background variabilities. In Fig. 4, the ROI size is fixed (ROI corresponding to the NEMA 5.6 ml sphere), and the metrics are plotted as a function of the iteration number. In Fig. 5, the iteration number is fixed at 2, and the metrics are plotted as a function of the ROI size. Each plot includes the four post-filtering options.

For the 5.6 ml sphere, Butterworth filter and no filter (none) give a significantly higher number of counts detected and sensitivity regardless of the iteration number (Fig. 4a, b). The sensitivity was maximal with two iterations regardless of the filter. These results applied for all the sphere sizes (Fig. 5a, b). Although these two configurations (Butterworth filter and no filter) presented the highest background counts (Fig. 4c), they provide a significantly higher SNR for the 5.6 ml sphere (Fig. 4d), and this result applied for all sphere dimensions, except for the biggest one (Fig. 5d). This metric was maximal with two iterations. The SNR increased with the hot sphere size (Fig. 5d), indicating better detection performance for larger objects, as expected.

**Fig. 6:**
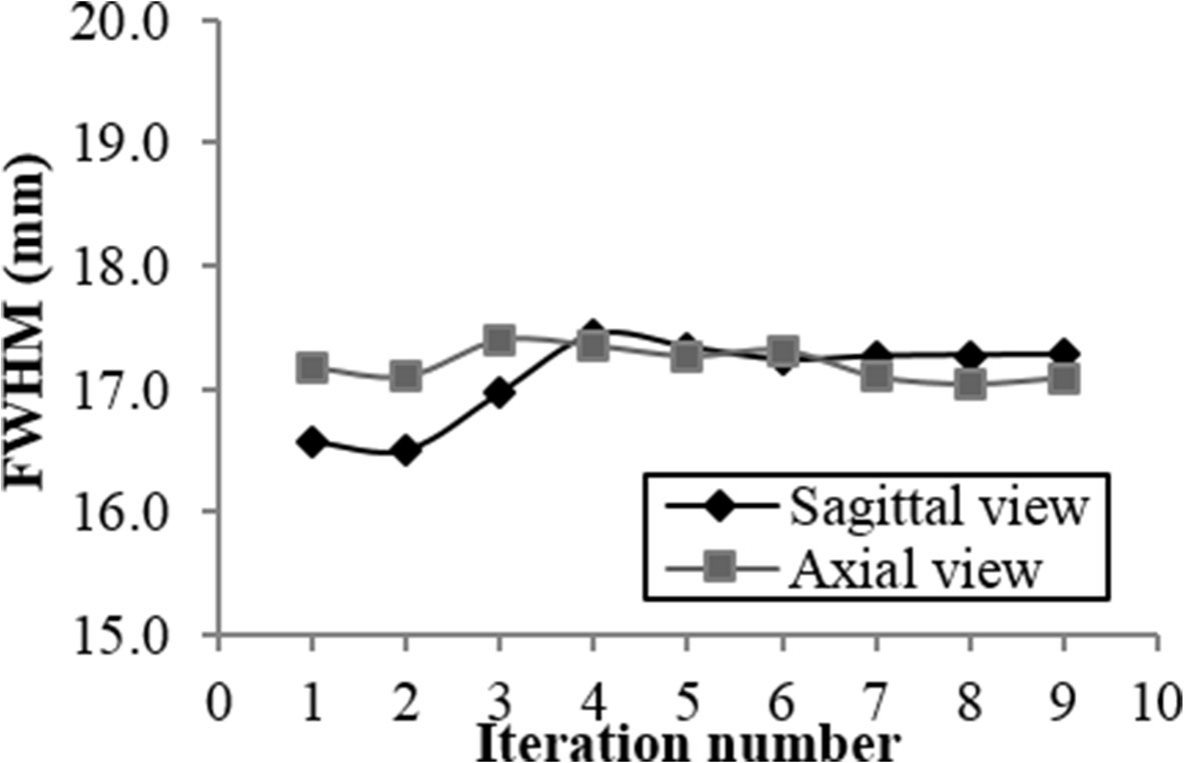
Spatial resolution measured in the Triple Line phantom plotted against iteration number

Figure 6 shows the evolution of the spatial resolution with the number of iterations on both axial and sagittal views. During the SPECT/CT reconstruction, the Butterworth filter was used. The results confirm that the FWHM increases with the number of iterations and justify the choice of two iterations. The spatial resolution obtained with these parameters is then 17.1 mm and 16.5 mm on the axial and sagittal views, respectively. This parameter is important because it determines the partial volume effect. The partial volume effect is a major limitation in quantification studies because it degrades activity determination mostly in structures having dimensions less than 3 × FWHM of the reconstructed images [28]. This might happen, in our case, for spherical structures with a radius smaller than 18 mm, which corresponds to a volume of 24 ml.

In summary, the most optimized reconstruction parameters included the use of the Butterworth filter and of a number of iterations equal to 2. The Butterworth filter was preferred over no filter because it improves the visual quality of the image, so the uptakes detection. These reconstruction parameters offered the best compromise between achieving maximum signal and minimum background noise. These parameters were used in the following experiments.

### Calibration factors

**Fig. 7:**
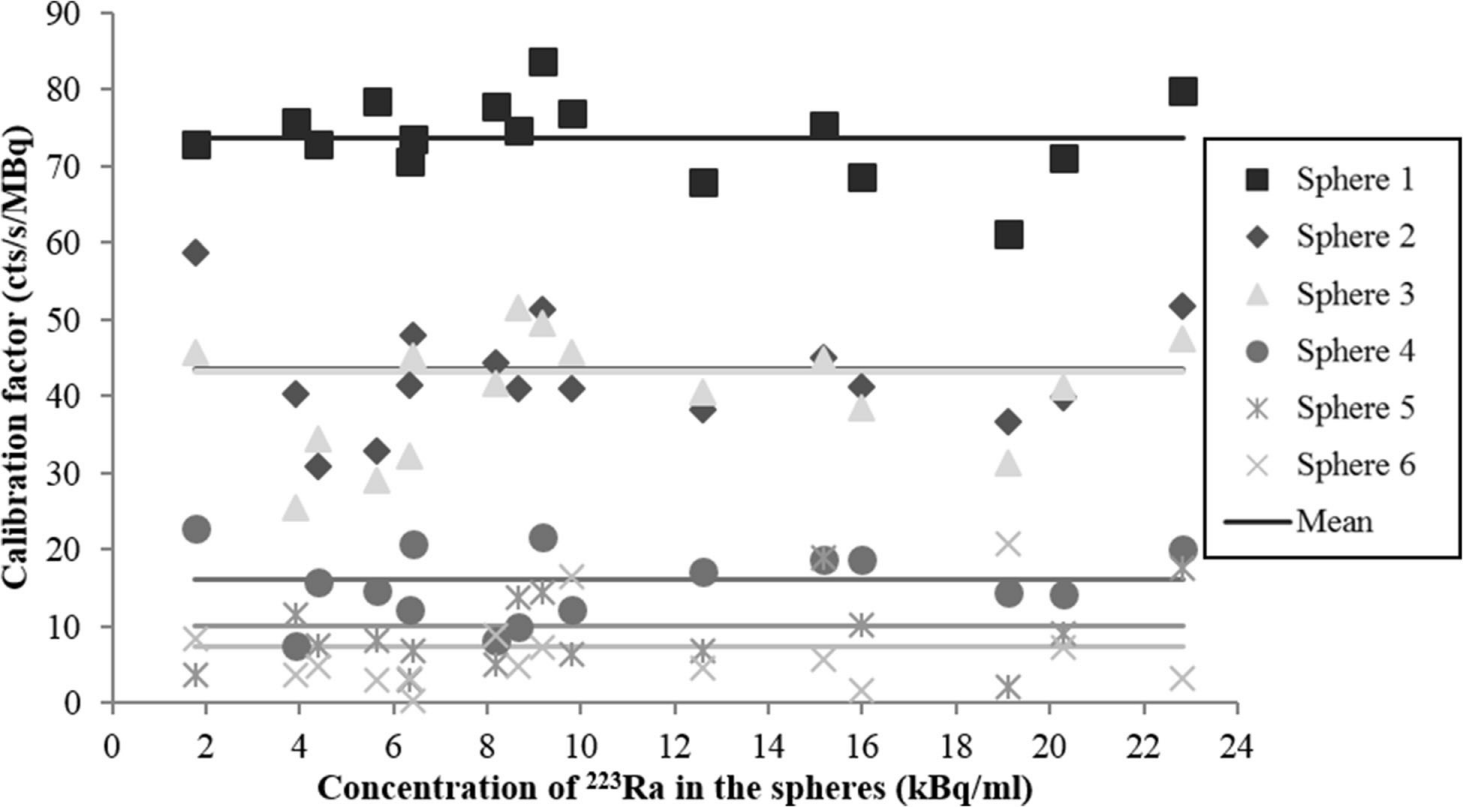
Evolution of the calibration factor with the concentration of ^223^Ra in each sphere of the NEMA phantom on SPECT/CT images

Figure 7 shows the dependence of the calibration factor (CF) with the concentration of ^223^Ra for each hot sphere of the NEMA phantom. Our results show that CF was constant for the three biggest spheres (spheres 1–3 in Fig. 7) with ^223^Ra concentration between 6.5 kBq/ml and 22.8 kBq/ml. In this concentration range, the calibration factor was 73.7 ± 6.2 cts/s/MBq (mean ± standard deviation) for the 26.5 ml sphere (sphere 1 in Fig. 7), 43.8 ± 5.1 cts/s/MBq for the 11.5 ml sphere (sphere 2 in Fig. 7), and 43.4 ± 5.6 cts/s/MBq for the 5.6 ml sphere (sphere 3 in Fig. 7). The difference between the calibration factors was 41% between the 26.5 ml and the 11.5 ml spheres, and 52% between the 26.5 ml and the 5.6 ml spheres. This is also explained by the partial volume effect as its impact increases when the volume decreases. The very similar values of the CF between 11.5 ml and 5.6 ml spheres can be explained by a very similar impact of the partial volume effect on these two volumes. Finally, for the three smaller spheres, the partial volume effect impact is higher and the quantification is further underestimated.

For lower activity concentrations, between 1.8 kBq/ml and 6.5 kBq/ml, the calibration factor, except from the biggest sphere (26.5 ml), exhibited greater variability. This was due to the partial volume effect which limited the detection and quantification.

### Validation with an anthropomorphic phantom

**Fig. 8:**
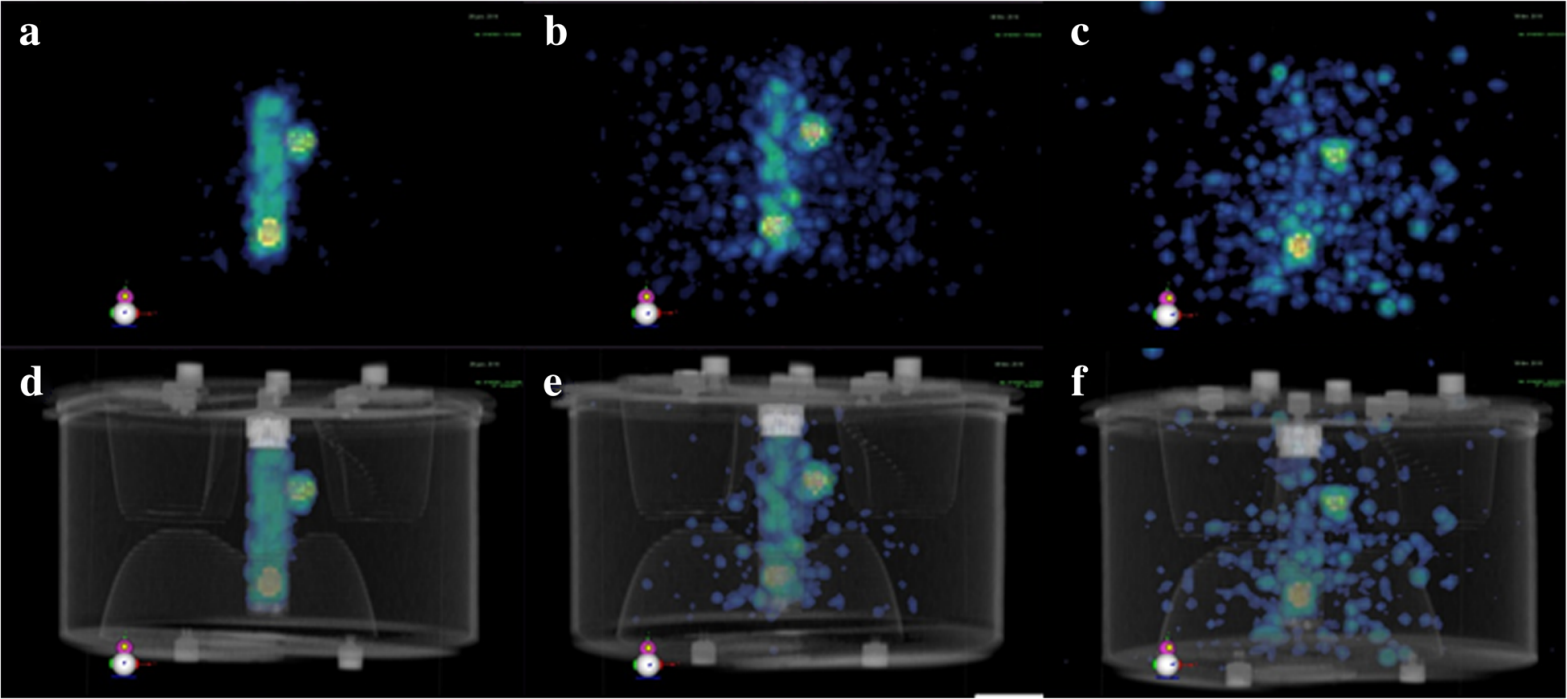
3D views of reconstructed SPECT/CT images of the TORSO phantom for three ^223^Ra concentrations and TNT ratios. **a** 27.5 kBq/ml, TNT = 6. **b** 14.9 kBq/ml, TNT = 10. **c** 8.1 kBq/ml, TNT = 30. The corresponding SPECT/CT fused images are shown in **d**, **e**, and **f**

Figure 8 shows some SPECT/CT images for three different TNT ratios. Despite the highest TNT ratios, Fig. 8c, f presented the biggest background because of the low activity in the inserts.

**Fig. 9:**
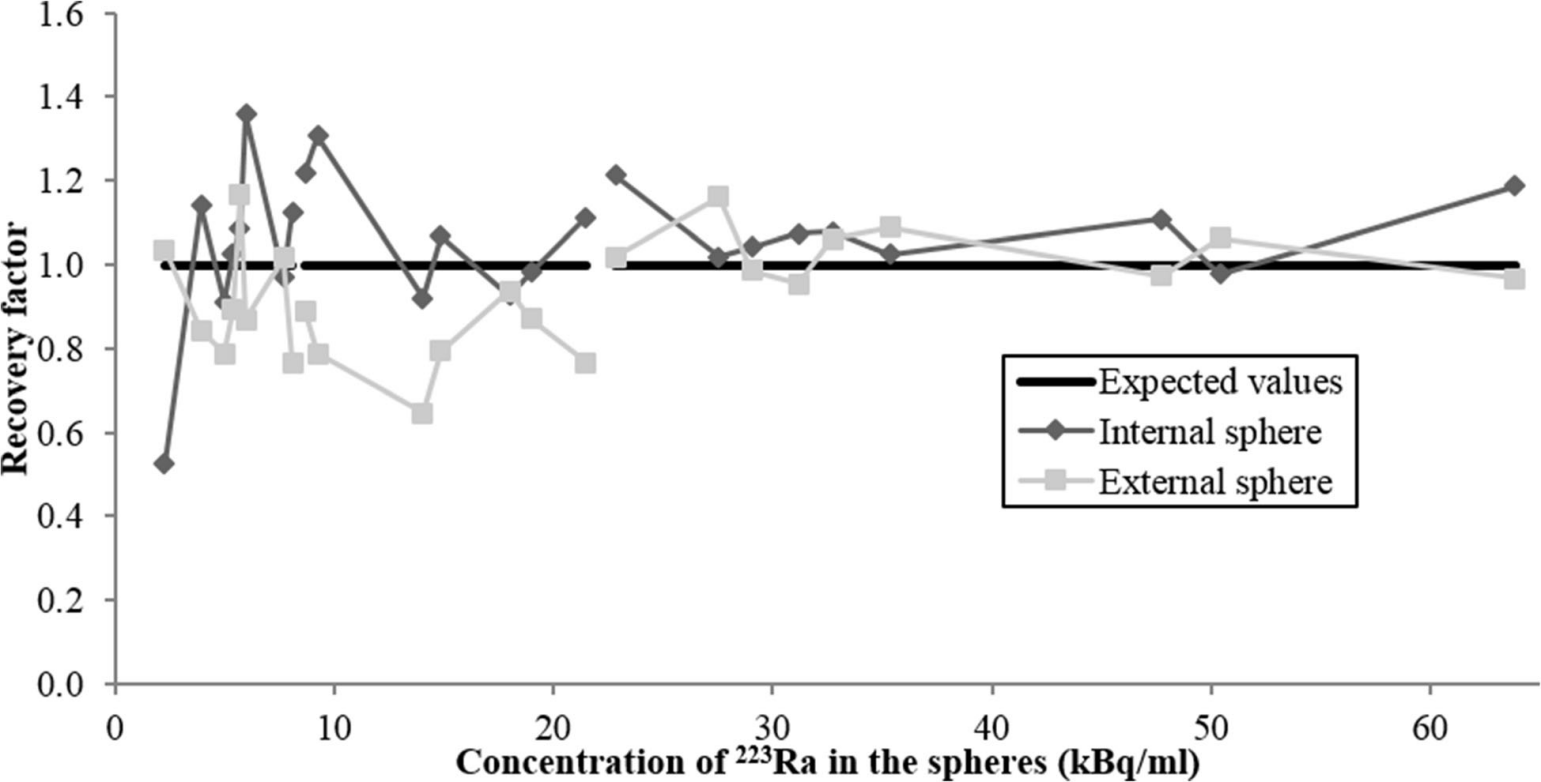
^223^Ra recovery factor as a function of ^223^Ra concentrations in both internal and external spheres

Figure 9 presents the ^223^Ra activity recovery in the two biggest spheres (5.6 ml) of the TORSO phantom as a function of the ^223^Ra concentration and TNT ratios. The theoretical (ground truth) value of the recovery factor is 1. The recovery factor (RF) variability around the ground truth increased inversely with ^223^Ra concentration. For ^223^Ra concentrations higher than 8 kBq/ml, RF calculated for the two spheres slightly varied around 1. For the internal sphere, RF was approximately 1.01 ± 0.09 (mean ± standard deviation) for a TNT ratio of 6 and 1.07 ± 0.14 for a TNT ratio of 10. For the external sphere, RF was approximately 0.96 ± 0.05 (TNT = 6) and 0.81 ± 0.09 (TNT = 10). However, for ^223^Ra concentrations lower than 8 kBq/ml, the RF presented a higher variability albeit it employed a higher TNT ratio. This finding was consistent with the results obtained from the NEMA phantom, where for the lowest concentration (2.3 kBq/ml), the internal sphere was faintly detectable (Fig. 8c, d).

The activity in the internal sphere was mainly overestimated, whereas the activity in the external sphere was mainly underestimated. This difference was probably due to the activity present in the background of the internal sphere, absent in the external sphere, that contributes to the so-called spill-in and spill-out effect [28].

In summary, this protocol and its validation show that activity could be quantified with a relative error of 1.1% for TNT = 6 and with a relative error of 6.7% for TNT = 10, for the internal sphere of 5.6 ml and for ^223^Ra concentrations greater than 8 kBq/ml. For the external sphere of 5.6 ml, the activity could be quantified with a relative error of − 4.0% for TNT = 6 and with a relative error of − 18.8% for TNT = 10, for ^223^Ra concentrations greater than 8 kBq/ml.

## Discussion

In this study, we established and optimized a protocol for ^223^Ra SPECT/CT acquisition and reconstruction for clinical use.

Three energy emission windows (85.0 keV ± 20%, 154.0 keV ± 10%, and 270.0 keV ± 10%) and three scatter windows (47.0 keV to 67.0 keV, 103.0 keV to 123.0 keV, and 210.2 keV to 242.8 keV) are recommended. Although Owaki et al. [24] used a single energy window at 84 keV ± 20%, we precise that the gamma camera used in our study is equipped with a 5/8-in. crystal. This crystal gives a better sensitivity than the more common 3/8-in. crystal. This percentage loss of sensitivity increases with rising photon energy [29]: the loss in sensitivity can approach 20% at 140 keV and 35% at 190 keV. We found that our crystal allow the use of the sum of the images from three energy windows without loss of spatial resolution. Our study with the NEMA phantom enabled the optimization of the reconstruction parameters (OSEM algorithm with 2 iterations, 10 subsets, and the Butterworth filter) yielding the best compromise between sensitivity and noise.

Our NEMA phantom study showed a constant calibration factor in the reconstructed images for the three largest spheres of 37 mm, 28 mm, and 22 mm diameter, corresponding to 26.5 ml, 11.5 ml, and 5.6 ml respectively, and with ^223^Ra concentrations from 6.5 kBq/ml to 22.8 kBq/ml. This calibration factors for the three biggest spheres of the NEMA phantom allow to adapt the calibration factor for different lesion dimensions [30]. For smaller spheres, the visual contrast and sensitivity decreased due to partial volume effects. In Pacilio et al. [16] and Murray et al. [31], the authors reported an average lesion size of 87 ml (1.2 to 270 ml in 14 patients and 53 lesions) for osteoblastic bone metastasis of prostate cancer. However, in the case of osteolytic bone metastasis of kidney cancer, which is the target of new clinical trials [32], lesions are much smaller (average volume of 0.6 ml specifically, 0.1 ml to 5.1 ml in 10 patients and 66 lesions) [33]. Thus, we predict that our protocol will enable the quantification of osteoblastic bone metastasis (with a significant uptake) whereas, for osteolytic bone metastasis, partial volume correction will be necessary to obtain a more robust quantification.

In our NEMA phantom study, we were also able to calibrate the gamma camera for each sphere size. The calibration method was validated under more realistic conditions using the TORSO phantom. The application of phantom-based results in clinical cases is far from being straightforward. Nevertheless, in this study, we tried to simulate clinical conditions as close as possible. Indeed, an anthropomorphic phantom was used, and the activity concentration ranges were close to clinical values [15]. In practice, any uptake volumes in patients will be approximate to a sphere (longest axis) in order to choose the best calibration factor based on the preliminary phantom’s study. This method will give better results than using fixed calibration factors that are usually applied in clinical routine. In these conditions and as a result of our optimization process, activity quantification was validated for structures bigger than 5.6 ml and for concentrations higher than 8 kBq/ml, with a residual error lower than 18.8%. Only one communication reported a quantitative study on ^223^Ra SPECT/CT imaging [34]. Based on a phantom study, the authors report a quantification error of 27% for a 22.8 ml sphere containing 5.1 kBq/ml and a TNT ratio of 30. These results are coherent with our experimental observations, which predict quantification errors greater than 18.8% for ^223^Ra concentrations lower than 8 kBq/ml.

Finally, in this study, we optimized the reconstruction protocol on a clinical software. However, precise reconstruction methods have to take into account the scatter and attenuation of ^223^Ra emissions in the system matrix of the iterative reconstruction algorithm by performing Monte Carlo simulations. Unfortunately, clinical software are not suited for the multiple photon emission of ^223^Ra. Thus, additional studies using Monte Carlo simulations, in particular GATE [35], should be carried out to more precisely reconstruct ^223^Ra SPECT/CT images and thus more precisely quantify ^223^Ra uptakes.

Interestingly, some studies are focusing on the correlation between uptakes of ^99m^Tc-labeled bone tracers (used for bone scintigraphy) or ^18^F-fluoride (^18^F-FNa used for positron emission tomography or PET) with ^223^Ra uptakes. Pacilio et al. [16] demonstrated a significant correlation between uptakes of ^223^Ra and ^99m^Tc in osteoblastic lesions. Similarly, Murray et al. [31, 36] observed a correlation not only between the uptakes of ^223^Ra and ^18^F-fluoride in the osteoblastic lesions but also with the absorbed dose as well. Therefore, it might be possible to predict the response of treatment in osteoblastic lesions by bone scintigraphy or PET imaging with ^18^F-fluoride. Although the uptakes of ^223^Ra and ^18^F-fluoride at the osteoblastic lesions may be the same, this is not necessarily the case for healthy tissues and elimination routes, suggesting a crucial role for ^223^Ra images. Moreover, the correlation between ^223^Ra and ^99m^Tc or ^18^F-fluoride in osteolytic lesions is yet to be proved. Finally, some lesions may preferentially bind to ^99m^Tc-labeled bone tracers or ^18^F-FNa rather than ^223^Ra [14, 36].

## Conclusion

Our phantom study showed that quantitative ^223^Ra SPECT/CT imaging using the predetermined calibration factors appears feasible under the specific condition where the dimension of the target is clearly known. The quantitation was susceptible to the concentration of ^223^Ra in the target, although its error seemed acceptable at clinically relevant levels of administered radioactivity. In addition, attenuation and scatter corrections in quantitative ^223^Ra imaging need further investigations. These results suggest that ^223^Ra SPECT/CT should be used to acquire images for therapeutic purposes in addition to currently acquired images for diagnostic purposes.

Accurate activity quantification is crucial to calculate the absorbed dose in organs and lesions, and might enable personalized absorbed dose-based treatment planning. We hope that this study will support future biodistribution, pharmacokinetic, and dosimetry studies aimed at establishing the relationship between the absorbed dose and the therapeutic response.

## Abbreviations

BV: Background variability
CF: Calibration factor
CRPC: Castration-resistant prostate cancer
FWHM: Full width at half maximum
GE: General Electric
LEGP: Low-energy general-purpose
LEHR: Low-energy high resolution
MEGP: Medium-energy general-purpose
MIRD: Committee on Medical Internal Radiation Dose
OSEM: Ordered subset expectation maximization
RF: Recovery factor
ROI: Regions of interest
SPECT: Single-photon emission computed tomography
SPECT/CT: Single-photon emission computed tomography/computed tomography
TNT: Tumor to normal tissue
